# Potentiation of cGMP signaling increases oxygen delivery and oxidative metabolism in contracting skeletal muscle of older but not young humans

**DOI:** 10.14814/phy2.12508

**Published:** 2015-08-13

**Authors:** Michael Nyberg, Peter Piil, Jon Egelund, Randy S Sprague, Stefan P Mortensen, Ylva Hellsten

**Affiliations:** 1 Department of Nutrition, Exercise and Sports, University of CopenhagenCopenhagen, Denmark; 2 Department of Pharmacological and Physiological ScienceSaint Louis, Missouri, USA; 3 Department of Cardiovascular and Renal Research, University of Southern DenmarkOdense, Denmark; 4 The Centre of Inflammation and Metabolism and the Centre for Physical Activity Research, Department of Infectious Diseases, Rigshospitalet, University of CopenhagenCopenhagen, Denmark

**Keywords:** Blood flow, exercise, sildenafil

## Abstract

Aging is associated with progressive loss of cardiovascular and skeletal muscle function. The impairment in physical capacity with advancing age could be related to an insufficient peripheral O_2_ delivery to the exercising muscles. Furthermore, the mechanisms underlying an impaired blood flow regulation remain unresolved. Cyclic guanosine monophosphate (cGMP) is one of the main second messengers that mediate smooth muscle vasodilation and alterations in cGMP signaling could, therefore, be one mechanism by which skeletal muscle perfusion is impaired with advancing age. The current study aimed to evaluate the effect of inhibiting the main enzyme involved in cGMP degradation, phosphodiesterase 5 (PDE5), on blood flow and O_2_ delivery in contracting skeletal muscle of young and older humans. A group of young (23 ± 1 years) and a group of older (72 ± 2 years) male human subjects performed submaximal knee-extensor exercise in a control setting and following intake of the highly selective PDE5 inhibitor sildenafil. Sildenafil increased leg O_2_ delivery (6–9%) and leg O_2_ uptake (10–12%) at all three exercise intensities in older but not young subjects. The increase in leg O_2_ delivery with sildenafil in the older subjects correlated with the increase in leg O_2_ uptake (*r*^2^ = 0.843). These findings suggest an insufficient O_2_ delivery to the contracting skeletal muscle of aged individuals and that reduced cGMP availability is a novel mechanism underlying impaired skeletal muscle perfusion with advancing age.

## Introduction

Life expectancy has steadily increased over the past two centuries, resulting in an increasing number of older individuals (Oeppen and Vaupel 2002). As aging is associated with progressive loss of cardiovascular and skeletal muscle function that often leads to progressive disability (Doherty 2003) and increased risk of cardiovascular disease (Buchner 2009), a better understanding of the mechanisms that contribute to the age-related changes in vascular and skeletal muscle function is needed.

Oxidative metabolism is the dominant source of energy for skeletal muscle and to ensure sufficient availability of O_2_, blood flow, and O_2_ delivery to the contracting muscles are closely regulated to match the O_2_ demand (Andersen and Saltin 1985; Saltin et al. 1998; Roach et al. 1999; Gonzalez-Alonso et al. 2001). Aging has consistently been reported to be associated with reduced blood flow and O_2_ delivery to the exercising limb (Wahren et al. 1974; Proctor et al. 1998, 2003; Lawrenson et al. 2003; Poole et al. 2003; Kirby et al. 2012; Nyberg et al. 2012) but the mechanisms underlying the altered regulation of exercise hyperemia have not been resolved (Proctor and Parker 2006). Furthermore, to what extent a reduced blood flow and O_2_ delivery to contracting skeletal muscle of older individuals have metabolic and functional consequences remain unclear (Proctor and Moore 2012). Blood flow to skeletal muscle is determined by perfusion pressure and vascular tone of which the latter is the result of the balance between vasoconstrictor and vasodilator signaling pathways in vascular smooth muscle cells (VSMC). Among these regulatory mechanisms is the cyclic nucleotide cyclic guanosine monophosphate (cGMP), which mediates its effects via activation of protein kinase G (PKG), is considered one of the main second messengers that mediate vasodilation (Morgado et al. 2012). Intracellular cGMP is degraded by cGMP-binding phosphodiesterase 5 (PDE5), and concentrations of cGMP are tightly controlled by this enzyme (Rybalkin et al. 2003). Sildenafil has been shown to specifically inhibit the catalytic site of PDE5 with a very high selectivity for PDE5 and this compound can, therefore, be used to potentiate cGMP signaling via inhibition of cGMP degradation (Ballard et al. 1998; Corbin et al. 2003). This effect of sildenafil on cGMP signaling in the vasculature can be assessed in vivo by infusion of the endothelium-dependent vasodilator ACh and the nitric oxide (NO) donor sodium nitroprusside (SNP) (endothelium-independent vasodilator) as the vasoactive effects of these compounds are mediated via formation of cGMP (Hellsten et al. 2012).

Evidence in the literature indicates that cGMP signaling may be impaired in aging. In older rats, the ability of cGMP to stimulate PKG in VSMC has been shown to be blunted compared to VSMC of young rats (Lin et al. 2001). Furthermore, the bioavailability of NO, which mediates its vasodilator effect via production of cGMP, is reduced in aging (Taddei et al. 2001; Nyberg et al. 2012). To what extent alterations in cGMP signaling are involved in the reduced blood flow and O_2_ delivery to exercising skeletal muscle in aging humans remain unknown.

We tested the hypothesis that potentiation of cGMP signaling would increase blood flow, O_2_ delivery and O_2_ uptake in contracting skeletal muscle of older but not young human subjects during submaximal exercise engaging a small muscle mass. To accomplish this, we examined the effect of the highly selective PDE5 inhibitor sildenafil on central and peripheral hemodynamics during small muscle mass exercise in a group of young and older subjects matched for physical activity level.

## Methods

A total of 17 healthy male subjects of which 9 were young (23 ± 1 years) and 8 were older (72 ± 2 years) participated in the study (Table1). The subjects underwent screening by means of a medical examination, 12-lead electrocardiogram and blood sampling from the antecubital vein. Exclusion criteria were history or symptoms of cardiovascular disease, renal dysfunction, insulin resistance, diabetes, or hypercholesterolemia. All subjects were nonsmokers and none of the subjects were taking prescription medicine. The study was approved by the Ethics Committee of Copenhagen (H-3-2012-176) and conducted in accordance with the guidelines of the *Declaration of Helsinki*. Written informed consent was obtained from all subjects before enrollment into the study.

**Table 1:** Subject characteristics

	Young	Older
Age (years)	23 ± 1	72 ± 2*
Height (m)	1.83 ± 0.01	1.80 ± 0.02
Body weight (kg)	75.8 ± 3.3	79.3 ± 4.8
Body fat (%)	13.7 ± 1.4	25.6 ± 1.4*
Systolic blood pressure (mmHg)	117 ± 3	138 ± 5*
Diastolic blood pressure (mmHg)	60 ± 1	62 ± 3
Mean arterial blood pressure (mmHg)	80 ± 2	90 ± 2*
Vo_2max_ (l/min)	3.42 ± 0.24	2.42 ± 0.12*
Vo_2max_ relative to body weight (l/min/kg)	45.9 ± 2.7	30.6 ± 0.8*
Experimental leg mass (kg)	11.9 ± 0.6	11.7 ± 0.4
Experimental lean leg mass (kg)	10.0 ± 0.5	8.9 ± 0.2
Peak workload during knee-extensor exercise (W)	48 ± 3	37 ± 2*
HbA1c (mmol/mol)	32 ± 1	37 ± 1*
Glucose, average (from HbA1c) (mmol/l)	5.4 ± 0.1	6.1 ± 0.1*
Total cholesterol (mmol/l)	4.3 ± 0.3	5.4 ± 0.3*
HDL-C (mmol/l)	1.4 ± 0.1	1.7 ± 0.2
LDL-C (mmol/l)	2.6 ± 0.2	3.2 ± 0.2*

Values are means ± SEM. Significant difference from young

* *P* < 0.05.

### Initial testing

Before the experimental day the subjects visited the laboratory to become accustomed to the one-leg knee-extensor model (Andersen and Saltin 1985) and to perform an incremental bicycle ergometer exercise test in which pulmonary maximal oxygen uptake (L min^−1^, 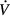
o_2max_) was determined (Oxycon Pro, Intramedic, Gentofte, Denmark; Table1). An incremental test was also performed in a one-leg knee-extensor ergometer to determine maximal workload (*W*_max_).

### Experimental protocol

Subjects refrained from caffeine, alcohol, and exercise for 24 h before the experimental day. On the day of the experiment the subjects arrived at the laboratory after eating breakfast. After local anesthesia (lidocaine, 20 mg mL^−1^; Astra Zeneca, Copenhagen, Denmark), catheters (20 Ga.; Arrow Int., Reading, PA) were placed in the femoral artery and vein of the experimental leg (right) and in the femoral artery of the nonexperimental leg (left). Following 30 min of rest, subjects were positioned in a supine position where they received femoral arterial infusion of: (1) ACh (25 and 100 *μ*g min^−1^ kg leg per mass; Miochol-E, Bausch & Lomb Inc., Bridgewater, NJ) and (2) SNP (4 *μ*g min^−1^ kg leg per mass; Nitropress, Hospira Inc., Lake Forest, IL). Each dose of ACh and SNP was infused for 2.5 min and measurements (blood flow, blood pressure and arterial and venous blood samples [∼2 mL]) were obtained after 2.0 min. Infusion of SNP was performed 30 min after infusion of the last dose of ACh was terminated. After an additional 60 min of rest, subjects performed knee-extensor exercise at three different intensities: 6 W, 12 W and 40% of the maximal workload obtained in the incremental test (40% *W*_max_; 19 ± 1 and 15 ± 1 W: young and older). Each workload was sustained for 5 min and measurements were obtained after 3.5 min. Exercise performed at 6 and 12 W were completed consecutively whereas 40% *W*_max_ was performed after 5 min of rest. Following exercise, subjects received an oral dose of the PDE5 inhibitor sildenafil (100 mg; Actavis, Hafnarfjordur, Iceland). One hour after sildenafil intake, infusion of ACh and SNP and knee-extensor exercise were repeated in the same order as during the control condition.

### Measurements and calculations

Femoral arterial blood flow (FABF) was measured with ultrasound Doppler (Vivid E9; GE Healthcare, Brondby, Denmark) equipped with a linear probe operating at an imaging frequency of 11 MHz and Doppler frequency of 5.0 MHz and as previously described (Nyberg et al. 2014).

Intra-arterial and intra-venous pressure was monitored with transducers (Pressure Monitoring Set, Edwards Lifesciences, Irvine, CA) positioned at the level of the catheters. Blood gases, hemoglobin, glucose, and lactate were measured using an ABL800 FLEX analyzer (Radiometer, Bronshoj, Denmark). Total cholesterol, low-density lipoprotein (LDL-C), and high-density lipoprotein (HDL-C) were analyzed using an automatic analyzer using enzymatic kits (Cobas 8000, Roche, Hvidovre, Denmark), HbA1c using HPLC and noradrenaline using ELISA (Research ELISA, LDN, Nordhorn, Germany). Leg mass was calculated from whole-body dual-energy x-ray absorptiometry scanning (Prodigy; GE Healthcare), leg vascular conductance (LVC) as FABF/(mean femoral arterial pressure [FAP] − mean femoral venous pressure [FVP]), leg O_2_ delivery as arterial O_2_ content × FABF, leg O_2_ uptake as arteriovenous O_2_ difference × FABF, leg lactate release as arteriovenous lactate difference × FABF, change in leg lactate release as leg lactate release with sildenafil – leg lactate release in the control setting, change in leg O_2_ delivery as leg O_2_ delivery with sildenafil – leg O_2_ delivery in the control setting, change in leg O_2_ uptake as leg O_2_ uptake with sildenafil – leg O_2_ delivery in the control setting and difference in leg O_2_ delivery – leg O_2_ uptake was calculated by subtracting leg O_2_ uptake from leg O_2_ delivery.

### Statistical analysis

Specific hypothesis testing during rest, infusion, and exercise was performed with two-way repeated-measures analyses of variance. After a significant *F* test, pairwise differences were identified using a Student-Newman–Keuls post hoc test. Differences between young and older subjects at specific time points were assessed with unpaired *t*-tests and changes within each group with one-sample *t*-tests. Pearson correlation analysis was used to determine relations of interest. The number of subjects was selected on basis of detecting ∼10% differences in blood flow, O_2_ delivery, and O_2_ uptake within each group with intake of sildenafil, as these were the main outcomes of the study. Statistical significance was set at a priori at 0.05 and data are presented as means ± SEM.

## Results

### Arterial ACh infusion

**Figure 1:**
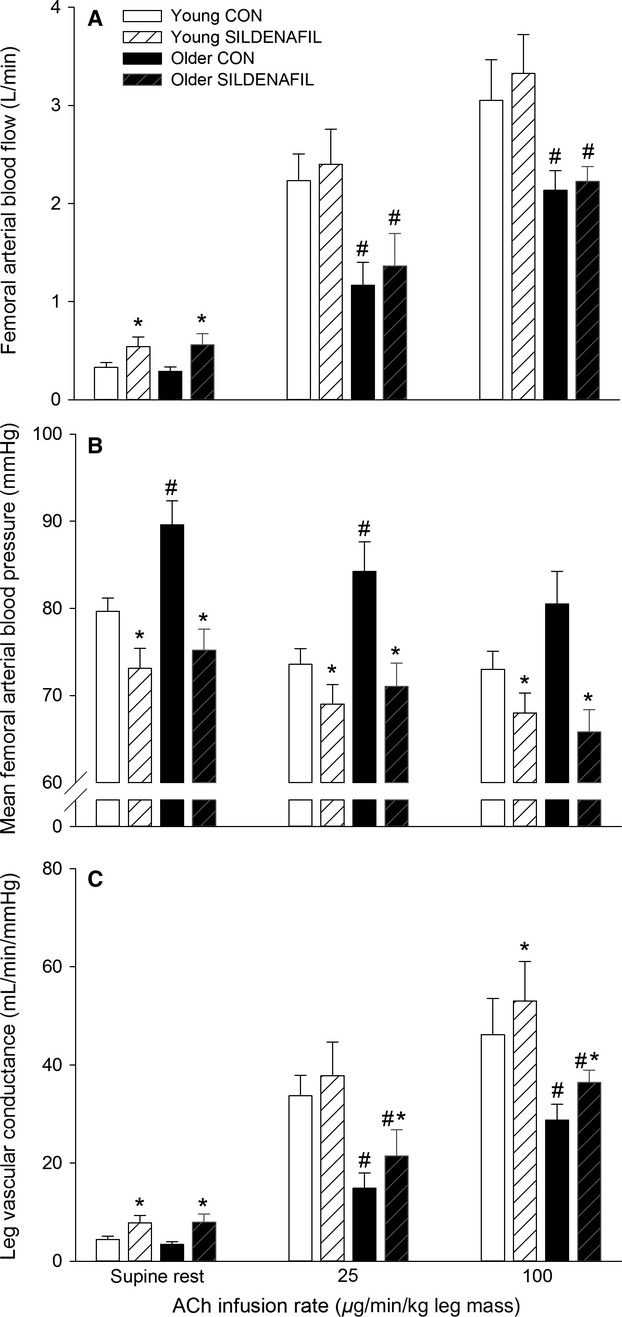
Femoral arterial blood flow (A), mean femoral arterial blood pressure (B), and leg vascular conductance (C) in young and older subjects at rest and during femoral arterial ACh infusion without (CON) and with sildenafil. Significant difference from CON within same condition: *P <* *0.05; significant difference from young within same condition: #*P *<* *0.05.

Sildenafil increased FABF at rest in both young (*P *<* *0.05) and older (*P *<* *0.05) subjects (Fig.1). In the older subjects, FABF was lower (*P *<* *0.05) than that of the young subjects during both infusion doses without and with sildenafil. FAP was lower with sildenafil at rest (*P *<* *0.05) and during infusion of the low (*P *<* *0.05) and high dose (*P *<* *0.05) of ACh. In the older subjects, FAP was higher (*P *<* *0.05) at rest and during infusion of the low dose of ACh, but this difference was abolished with sildenafil. At rest and during each infusion, FAP decreased to a larger extent (*P *<* *0.05) in the older (14 ± 2, 13 ± 2 and 15 ± 2 mmHg; rest, low dose and high dose) compared to the young (7 ± 2, 5 ± 2 and 5 ± 2 mmHg) subjects with sildenafil. At rest, femoral venous plasma noradrenaline increased to a similar extent in the young (1.3 ± 0.2 nmol L^−1^) and older (1.6 ± 0.7 nmol L^−1^) subjects with sildenafil. Following intake of sildenafil, LVC increased (*P *<* *0.05) at rest and during the highest dose of ACh in both groups whereas it only increased (*P *<* *0.05) in the older subjects during the low dose of ACh. LVC was lower (*P *<* *0.05) in the older subjects during both infusion doses with and without sildenafil. Blood variables, heart rate and FVP are presented in Table S1A and B.

### Arterial SNP infusion

**Figure 2:**
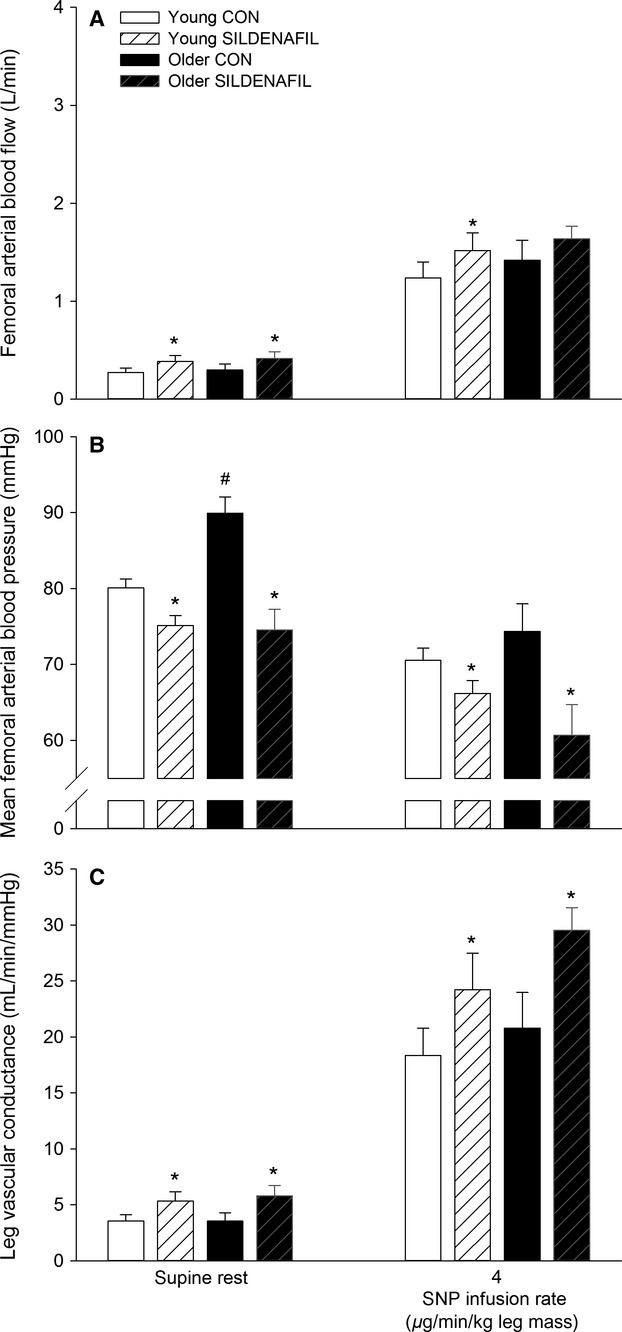
Femoral arterial blood flow (A), mean femoral arterial blood pressure (B), and leg vascular conductance (C) in young and older subjects at rest and during femoral arterial sodium nitroprusside (SNP) infusion without (CON) and with sildenafil. Significant difference from CON within same condition: *P <* *0.05; significant difference from young within same condition: #*P *<* *0.05.

Sildenafil increased (*P *<* *0.05) FABF during SNP infusion in the young subjects whereas no significant difference was detected in the older group (Fig.2). FAP was lower (*P *<* *0.05) with sildenafil during infusion of SNP in young and older subjects, resulting in higher (*P *<* *0.05) LVC in both groups during infusion of SNP. The decrease in FAP with SNP was more pronounced (*P *<* *0.05) in the older (14 ± 2 mmHg) compared to the young (4 ± 2 mmHg). A difference in the magnitude of change in FABF (0.17 ± 0.12 and 0.15 ± 0.14 L min^−1^, young and older subjects) and LVC (4.1 ± 1.9 and 7.3 ± 1.8 mL min^−1^ mmHg^−1^) with SNP without and with sildenafil was not detected between the young and older groups. Blood variables, heart rate, and FVP are presented in Table S2A and B.

### Knee-extensor exercise

**Figure 3:**
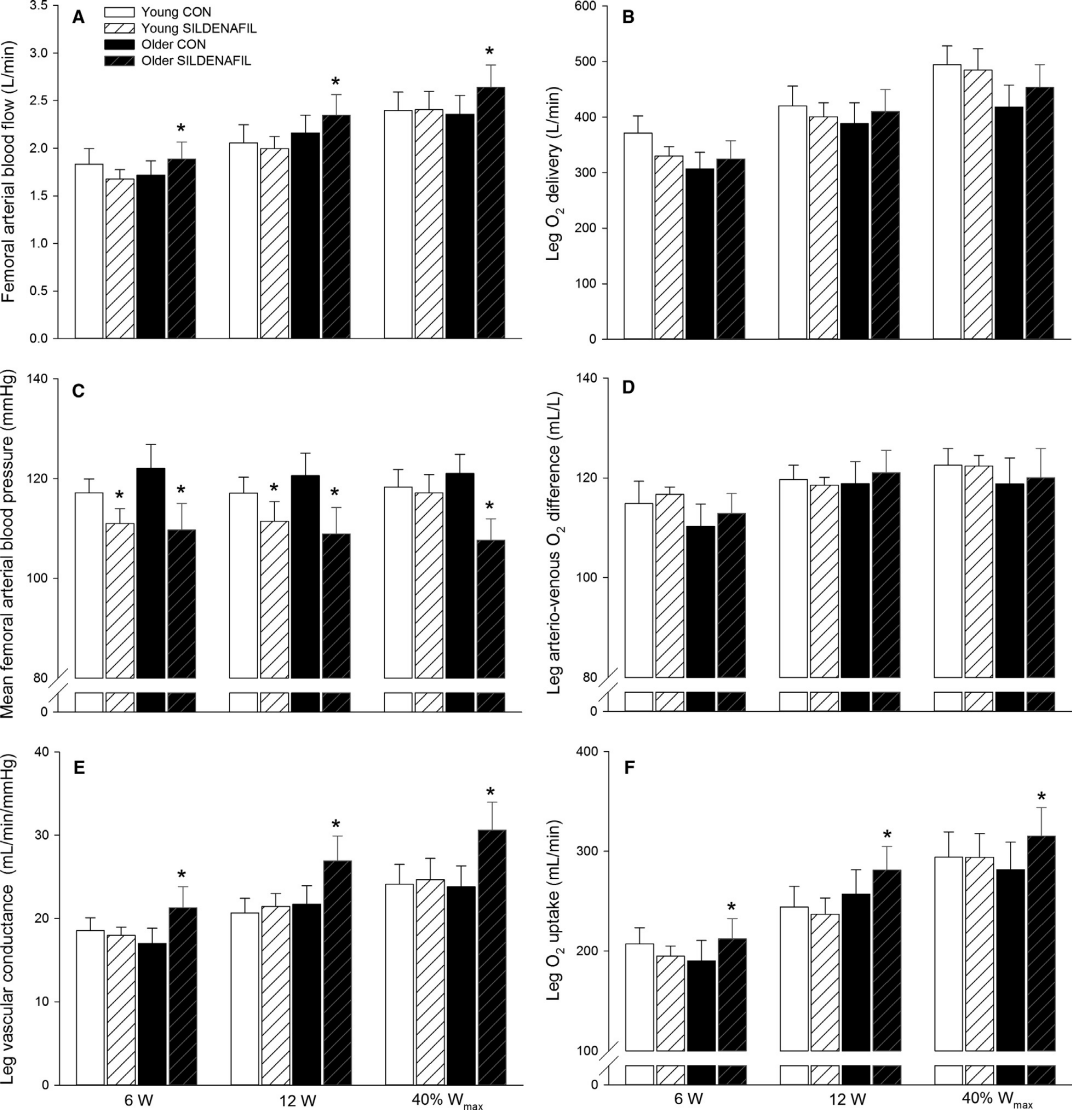
Femoral arterial blood flow (A), leg O_2_ delivery (B), mean femoral arterial blood pressure (C), leg arteriovenous O_2_ difference (D), leg vascular conductance (E), and leg O_2_ uptake (F) in young and older subjects during knee-extensor exercise performed at 6 W, 12 W, and 40% *W*_max_ (19 ± 1 and 15 ± 1 W: young and older) without (CON) and with sildenafil. Significant difference from CON within same condition: *P <* *0.05.

Sildenafil increased (*P *<* *0.05) FABF at all exercise intensities in the older subjects (Fig.3). Leg O_2_ uptake increased (*P *<* *0.05) at all intensities in the older group with sildenafil. In the young group, FAP was lower (*P *<* *0.05) with sildenafil at 6 and 12 W and lower (*P *<* *0.05) at all exercise intensities in the older group. LVC with sildenafil was unaltered in the young and increased (*P *<* *0.05) at all exercise intensities in the older subjects.

**Figure 4:**
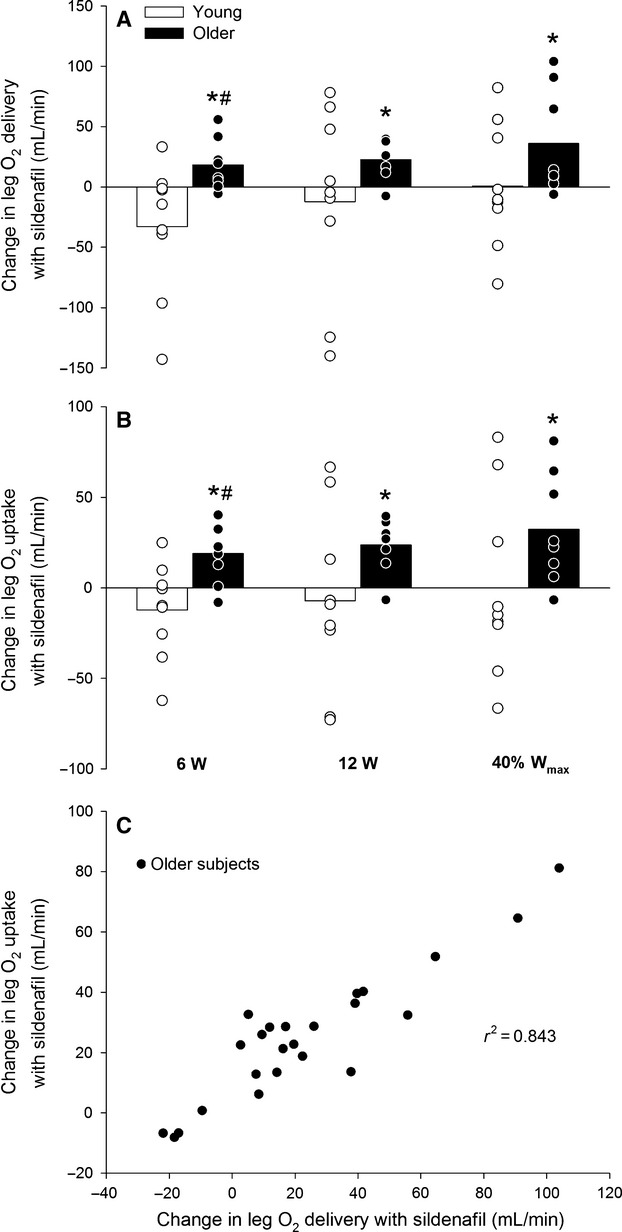
Change in leg O_2_ delivery with sildenafil (A), change in leg O_2_ uptake with sildenafil (B) and the association between the change in leg O_2_ delivery and the change in leg O_2_ delivery with sildenafil (C) in young and older subjects performing knee-extensor exercise at 6 W, 12 W, and 40% *W*_max_ (19 ± 1 and 15 ± 1 W: young and older). Significant difference: *P <* *0.05; significant difference from young within same condition: #*P *<* *0.05.

**Figure 5:**
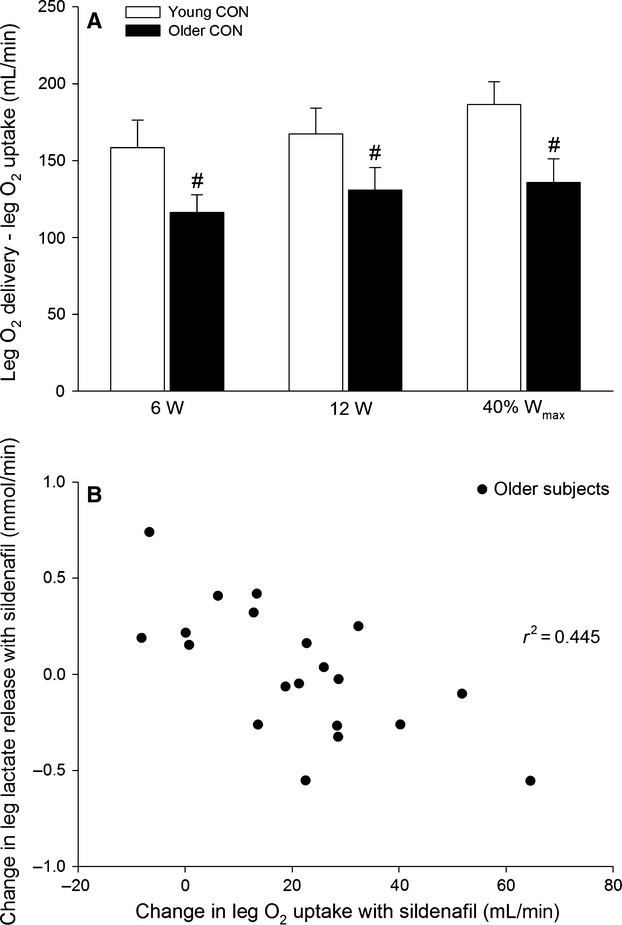
Difference between leg O_2_ delivery and leg O_2_ uptake (A) and association between change in leg O_2_ uptake and lactate release with sildenafil (B) in young and older subjects performing knee-extensor exercise at 6 W, 12 W, and 40% *W*_max_ (19 ± 1 and 15 ± 1 W: young and older). Significant difference from young within same condition: #*P *<* *0.05.

**Figure 6:**
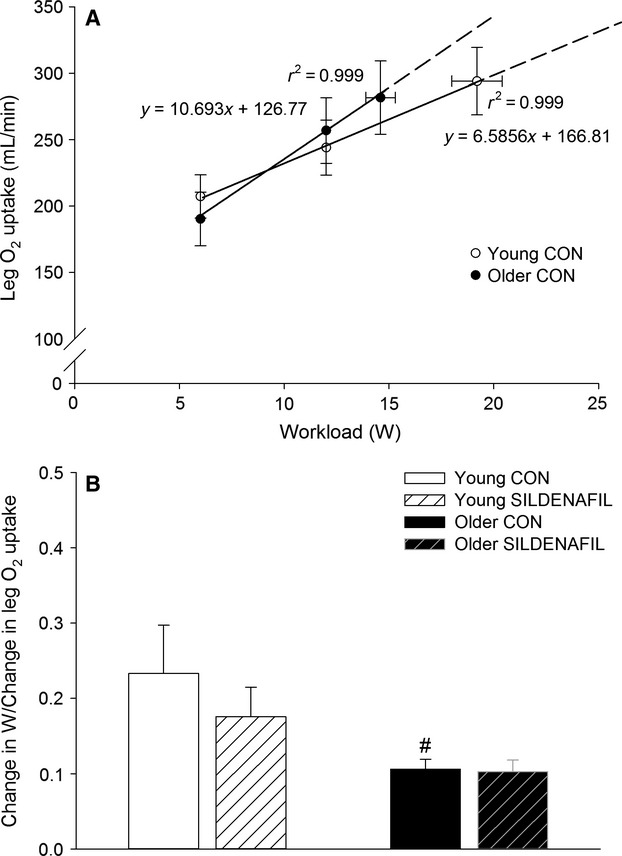
Association between workload and leg O_2_ uptake (A) and change in power output for a given change in leg O_2_ uptake (B) in young and older subjects performing knee knee-extensor exercise at 6 W, 12 W, and 40% *W*_max_ (19 ± 1 and 15 ± 1 W: young and older). Significant difference from young within same condition: #*P *<* *0.05.

When expressed as change in response to sildenafil intake, leg O_2_ delivery (6.3 ± 2.4, 6.0 ± 1.7 and 9.3 ± 4.0%; 6, 12 and 40% *W*_max_), and leg O_2_ uptake (11.6 ± 3.9, 10.4 ± 2.8 and 12.8 ± 4.8%) increased (*P *<* *0.05) at all exercise intensities in the older group (Fig.4). The change in leg O_2_ delivery with sildenafil correlated with the change in leg O_2_ uptake in the older (*r*^2^ = 0.843, *P *<* *0.001) but not young (*r*^2^ = 0.024) subjects. The difference between leg O_2_ delivery and O_2_ uptake was greater (*P *<* *0.05) in the young compared to the older subjects at all exercise intensities in the control condition and a negative association between the change in leg O_2_ uptake and leg lactate release was detected in the older (*r*^2^ = 0.445, *P* = 0.001) and young (*r*^2^ = 0.281, *P* = 0.004) subjects (Fig.5). The change in leg O_2_ uptake for a given change in power output during knee-extensor exercise (calculated as the slope of the linear regression of leg O_2_ uptake on power output for each subject; Fig.6A) was lower (*P *<* *0.05) in older compared to young subjects in the control condition (Fig.6B). No significant difference was detected in the change in power output for a given change in leg O_2_ uptake with sildenafil in either group. Blood variables, heart rate, and FVP are presented in Table2.

**Table 2:** Blood variables during knee-extensor exercise

Blood variable	6 W		12 W		40% *W*_max_	
	CON	SILD	CON	SILD	CON	SILD
**Young**						
PO_2_ (mmHg)						
a	107 ± 3	102 ± 2*	103 ± 3	102 ± 2	104 ± 2	103 ± 2
v	26 ± 1	25 ± 1	25 ± 1	25 ± 1	25 ± 1	25 ± 1
Hemoglobin (g dL^−1^)						
a	14.6 ± 0.2	14.5 ± 0.2	14.7 ± 0.2	14.7 ± 0.2	14.7 ± 0.3	14.7 ± 0.2
v	14.4 ± 0.3	14.2 ± 0.3	14.4 ± 0.2	14.1 ± 0.2	14.2 ± 0.3	14.2 ± 0.3
O_2_ saturation (%)						
a	98.2 ± 0.1	98.0 ± 0.1	98.1 ± 0.1	97.9 ± 0.1	98.1 ± 0.1	98.0 ± 0.1
v	40.7 ± 2.3	39.8 ± 1.2	38.9 ± 2.1	40.1 ± 1.4	38.2 ± 1.8	38.5 ± 1.5
O_2_ content (mL L^−1^)						
a	195 ± 2	194 ± 3	196 ± 3	195 ± 3	196 ± 3	196 ± 3
v	80 ± 6	77 ± 3	76 ± 5	77 ± 3	74 ± 5	74 ± 3
Lactate (mmol L^−1^)						
a	1.5 ± 0.3	1.2 ± 0.2	1.8 ± 0.4	1.3 ± 0.3	2.1 ± 0.4	1.6 ± 0.4
v	2.2 ± 0.5	1.5 ± 0.3*	2.4 ± 0.6	1.5 ± 0.4*	2.6 ± 0.6	2.1 ± 0.6
Lactate release (mmol min^−1^)						
	1.1 ± 0.3	0.5 ± 0.3*	1.2 ± 0.6	0.4 ± 0.4*	1.2 ± 0.7	1.1 ± 0.6
pH						
a	7.394 ± 0.007	7.393 ± 0.005	7.384 ± 0.006	7.396 ± 0.004	7.380 ± 0.005	7.394 ± 0.007*
v	7.304 ± 0.011	7.327 ± 0.005*	7.296 ± 0.012	7.320 ± 0.010*	7.289 ± 0.011	7.306 ± 0.012*
Heart rate (beats per minute)						
	89 ± 7	87 ± 5	94 ± 8	94 ± 6	99 ± 6	100 ± 6
FVP (mmHg)						
	18.4 ± 0.9	17.4 ± 1.2	17.6 ± 1.0	17.3 ± 1.0	17.5 ± 0.8	18.0 ± 1.0
**Older**						
PO_2_ (mmHg)						
a	85 ± 4†	76 ± 3*†	83 ± 3†	77 ± 3†	80 ± 4†	78 ± 4†
v	25 ± 1	23 ± 0^*^^*^	24 ± 1	22 ± 1	23 ± 1	22 ± 1
Hemoglobin (g dL^−1^)						
a	13.6 ± 0.3†	13.3 ± 0.4*†	13.7 ± 0.4†	13.5 ± 0.3†	13.5 ± 0.3†	13.3 ± 0.4†
v	13.4 ± 0.3†	13.1 ± 0.4†	13.3 ± 0.4†	13.0 ± 0.3†	13.6 ± 0.3	13.3 ± 0.3
O_2_ saturation (%)						
a	96.1 ± 0.3†	94.8 ± 0.4*†	95.9 ± 0.3†	94.9 ± 0.5*†	95.5 ± 0.5†	95.1 ± 0.6†
v	37.0 ± 1.8	33.1 ± 1.5*†	33.3 ± 1.7†	30.1 ± 2.2*†	31.2 ± 2.2†	28.5 ± 2.0†
O_2_ content (mL L^−1^)						
a	177 ± 4†	172 ± 5*†	178 ± 5†	174 ± 6*†	176 ± 4†	172 ± 5*†
v	67 ± 3†	59 ± 3*†	60 ± 3*†	53 ± 4†	57 ± 4†	51 ± 4*†
Lactate (mmol L^−1^)						
a	1.2 ± 0.2	1.0 ± 0.1	1.2 ± 0.1	1.1 ± 0.1	1.2 ± 0.1	1.1 ± 0.1
v	1.5 ± 0.2	1.2 ± 0.1	1.4 ± 0.2	1.2 ± 0.1	1.6 ± 0.2	1.4 ± 0.2
Lactate release (mmol min^−1^)						
	0.6 ± 0.1	0.4 ± 0.2	0.5 ± 0.2	0.3 ± 0.2	0.7 ± 0.3	0.7 ± 0.3
pH						
a	7.413 ± 0.006†	7.421 ± 0.005†	7.412 ± 0.005†	7.424 ± 0.006†	7.410 ± 0.008†	7.431 ± 0.005*†
v	7.336 ± 0.007†	7.350 ± 0.004*	7.337 ± 0.005†	7.342 ± 0.006	7.333 ± 0.006†	7.339 ± 0.006†
Heart rate (beats per minute)						
	74 ± 5	74 ± 5	78 ± 5	79 ± 6	82 ± 6	81 ± 6
FVP (mmHg)						
	19.7 ± 1.6	19.0 ± 1.8	19.7 ± 1.8	19.8 ± 1.9	20.2 ± 1.8	19.7 ± 1.9

a, femoral arterial; v, femoral venous.

* Significant difference from CON within same condition (*P* < 0.05).

† Significant difference from young within same condition (*P* < 0.05).

There were no associations between the changes in leg O_2_ delivery with sildenafil and age, systolic blood pressure, FAP, HbA1c, total cholesterol, and LDL cholesterol.

## Discussion

The findings from the current study demonstrate that potentiation of cGMP signaling by inhibition of PDE5 activity increases blood flow, O_2_ delivery, and O_2_ uptake in the exercising lower limb of older but not young human subjects during submaximal exercise engaging a small muscle mass. This effect of cGMP potentiation suggests an insufficient blood flow and O_2_ delivery to the contracting skeletal muscle of aged individuals and that a reduced cGMP level is a novel mechanism by which skeletal muscle blood flow and O_2_ delivery are impaired with advancing age in humans.

Blood flow to contracting skeletal muscle is closely regulated as a function of O_2_ bound to hemoglobin and arterial O_2_ content so that O_2_ delivery matches the O_2_ demand (Saltin et al. 1998; Roach et al. 1999; Gonzalez-Alonso et al. 2001). Aging is associated with a decline in hemoglobin levels and arterial O_2_ content (Ershler et al. 2005). Therefore, a lowered (Proctor et al. 1998; Lawrenson et al. 2003; Poole et al. 2003; Kirby et al. 2012; Nyberg et al. 2012) or unaltered (Beere et al. 1999; Proctor et al. 2003) blood flow to contracting skeletal muscle in aged individuals could result in an insufficient O_2_ delivery to meet the O_2_ demand of the contracting skeletal muscles. In the current study, hemoglobin levels, arterial O_2_ content, and the difference between O_2_ delivery and O_2_ uptake were lower in the older group, suggesting that O_2_ delivery could limit oxidative metabolism. We did not detect a difference in O_2_ uptake during submaximal exercise between the young and the older group in the control setting and although this would indicate that O_2_ delivery was sufficient to meet the O_2_ demand, age-related differences in mechanical efficiency with the possibility of anaerobic energy contribution needs to be taken into account. Accordingly, impaired mitochondrial and contractile efficiency has been documented in the human quadriceps muscle of aged subjects (Conley et al. 2013; Layec et al. 2015), suggesting that the metabolic demand for the same absolute workload was higher in the older group in the current study. The change in power output for a given change in leg O_2_ uptake during knee-extensor exercise was also found to be lower in the older group (Fig.6A and B), which is in support of a lower mechanical efficiency. Hence, the increase in O_2_ uptake with sildenafil in the older subjects is likely to reflect that oxidative metabolism in the control setting was compromised by insufficient O_2_ delivery. Notably, the lower difference between O_2_ delivery and O_2_ uptake in the older group was unaltered with sildenafil. This finding is explained by the close association between the increase in O_2_ delivery and O_2_ uptake and is in agreement with an insufficient O_2_ delivery in the control setting.

An insufficient O_2_ delivery to meet the oxidative demand in the older subjects would entail that anaerobic metabolism compensated for the lower aerobic metabolism. With regard to quantification of anaerobic metabolism, although lactate is formed and utilized continuously under fully aerobic conditions (Brooks 2009). Lactate leaving the exercising limb is still descriptive of anaerobic glycolysis in skeletal muscle (Juel and Halestrap 1999). As no difference in leg lactate release in the control condition was detected between the two groups, it may be that the intracellular level of lactate in the muscle of the older subjects was higher as the capacity to release lactate from the active muscle fibers may have been reduced. In line with this suggestion, the two important lactate transporters MCT1 and MCT4 have been shown to be downregulated in aging skeletal muscle (Masuda et al. 2009). Furthermore, in a previous study on older lifelong physically sedentary subjects, lactate release from the exercising leg was found to be similar to that of young adults despite a lower leg O_2_ uptake in the older group (Nyberg et al. 2012), indicating that lactate release was reduced as anaerobic metabolism would be expected to compensate for the lower oxidative metabolism. Importantly, the change in leg O_2_ uptake with sildenafil in the current study was negatively correlated with the change in lactate release, indicating that the increase in oxidative metabolism reduced anaerobic metabolism.

An insufficient O_2_ delivery to meet the metabolic demand of the contracting skeletal muscles in aging was also supported by a close association (*r*^2^ = 0.843) between the change in O_2_ delivery and O_2_ uptake with sildenafil. Alternatively, the increase in O_2_ delivery with sildenafil could be a result of an increased metabolic demand due to reduced mitochondrial efficiency, however, sildenafil has been shown not to affect oxidative phosphorylation in isolated mitochondria (Fernandes et al. 2008) and lactate levels and the change in power output for a given change in leg O_2_ uptake did not change with sildenafil.

In the current study, blood flow, O_2_ delivery, and O_2_ uptake was found to increase by ∼6–12% during exercise in the older subjects with sildenafil. Previous studies have shown reductions of ∼10–20% in blood flow and O_2_ uptake (Proctor et al. 1998; Lawrenson et al. 2003; Poole et al. 2003; Kirby et al. 2012; Nyberg et al. 2012) in aged individuals compared to young, indicating that the increase in these variables in the current study are of physiological relevance. It would, therefore, be of interest in future studies to examine the extent to which PDE5 inhibition can improve the functional capacity of older subjects. In this context, it may be that during exercise involving a large muscle mass, arterial blood pressure following sildenafil intake could compromise perfusion of the contracting muscles, thereby negating the effect observed during small muscle mass exercise.

Despite many efforts to determine the effects of aging on exercise hyperemia, a fundamental question regarding the mechanisms by which blood flow is altered remains unanswered. The cyclic nucleotide cGMP is considered one of the main second messengers that mediate vasodilation (Morgado et al. 2012). Acute potentiation of cGMP signaling via inhibition of PDE5 has been shown to increase blood flow following forearm contractions in hypertensive subjects (Attina et al. 2008) and skeletal muscle oxygenation during exercise in patients with atherosclerotic disease (Roseguini et al. 2014) and an altered cGMP signaling could be one mechanism by which blood flow regulation during exercise is impaired in aging. In the present study, potentiation of cGMP signaling by inhibition of PDE5 increased LVC and blood flow during exercise in the older but not young group. This effect of PDE5 inhibition specifically in the older group could reflect that aged individuals have generally higher activity of PDE5 and/or lower cGMP formation in contracting skeletal muscle. Although it is difficult to differentiate between these mechanisms based on an integrative response to PDE5 inhibition, the finding that sildenafil increased LVC to a similar extent in response to infusion of an NO donor is indicative of a similar activity of PDE5 in the young and older subjects. Hence, this finding on the vascular response to an NO donor is in line with a reduced cGMP formation in contracting skeletal muscle of older individuals.

The vasoactive substance NO plays a key role in the regulation of systemic blood pressure (Rees et al. 1989). Thus, the observation that sildenafil was found to induce a more marked reduction in blood pressure in the older subjects is in agreement with a reduced cGMP availability in the older subjects. This effect of PDE5 inhibition on blood pressure in the older group could be related to a higher initial blood pressure and hence less pronounced baroreceptor buffering in these subjects. However, a difference in the change in venous noradrenaline, which has been shown to correlate closely with acute changes in skeletal muscle sympathetic nervous activity (Grassi et al. 2008), was not found between the two groups following intake of sildenafil. This observation is also in line with a previous study demonstrating that the reduction in blood pressure with sildenafil was more related to age than blood pressure per se (Vardi et al. 2002).

It has been shown that aging is associated with a reduced vascular response to infusion of the endothelium-dependent vasodilator ACh (Taddei et al. 2001; Mortensen et al. 2012) as a consequence of a lower NO bioavailability (Taddei et al. 2001). In the current study, the vasodilator response to ACh was accordingly found to be lower in the older than the young group in the control setting. Although speculative, this could indicate that a diminished endothelial NO bioavailability was one factor contributing to lower cGMP levels during exercise. Notably, NO does not appear to be obligatory for exercise hyperemia during lower limb exercise in young subjects (Radegran and Saltin 1999) and a lower NO bioavailability would, therefore, entail an altered role of the NO system and/or that redundant systems that would normally compensate for a reduced NO formation are also affected by aging (Mortensen et al. 2007; Schrage et al. 2007).

Many biological events associated with advancing age are due to complex and integrated alterations in physiological systems that are influenced by genetic and life-style factors. One important lifestyle factor is the level of physical activity as evidenced by studies on enforced inactivity such as bed rest that initiates an “accelerated aging” process (Saltin et al. 1968; McGuire et al. 2001), and it has been argued that being physically active is the default requirement for maintaining health and physiological function throughout the life span (Lazarus and Harridge 2010). Therefore, we chose to include subjects that were characterized by a moderate level of physical activity in order to limit the potential adverse effects of skeletal muscle disuse in an attempt to elucidate the effects of primary aging. Hence, the finding that PDE5 inhibition increased blood flow and O_2_ uptake in these recreationally active subjects indicates that groups characterized by pronounced impaired endothelial function such as hypertension (Taddei et al. 1997; Attina et al. 2008), diabetes (Schalkwijk and Stehouwer 2005) and hypercholesterolemia (Creager et al. 1990) may benefit even more from potentiation of cGMP signaling.

## Summary

The current study shows that potentiation of cGMP signaling increases blood flow, O_2_ delivery, and oxidative metabolism in the exercising lower limb of older but not young healthy human subjects during submaximal exercise engaging only a small muscle mass. This finding suggest that a reduced O_2_ delivery to contracting skeletal muscle of older individuals have metabolic consequences that, at least in part, may explain the impairment in physical capacity associated with aging and that reduced levels of cGMP is one mechanism underlying the insufficient O_2_ delivery.

## Supporting Information

**Table S1.** Blood variables at rest and during ACh infusion. (A) Young, (B) older. **Table S2.** Blood variables at rest and during SNP infusion. (A) Young, (B) older.
