# Supplementary material for: Potentiation of cGMP signaling increases oxygen delivery and oxidative metabolism in contracting skeletal muscle of older but not young humans
Source: Physiol Rep. 2015 Aug 13;3(8):e12508. doi: 10.14814/phy2.12508 (PMC4562591; doi:10.14814/phy2.12508)
Supplement: Supplementary file 1 [file phy20003-e12508-sd1.pdf]

Online Table 1A. Blood variables at rest and during ACh infusion

	Young					
Blood variable	Rest		ACh 1		ACh 2	
	CON	SILD	CON	SILD	CON	SILD
PO2 (mmHg)						
a	105±3	108±3	104±2	106±2	104±2	106±2
v	39±1	45±2*	66±4	67±3	74±2	74±3
Hemoglobin (g dl-1)						
a	13.8±0.2	13.8±0.2	13.9±0.2	13.8±0.2	13.9±0.2	13.8±0.2
v	13.8±0.2	13.4±0.2	13.7±0.2	13.6±0.2	13.6±0.2	13.6±0.2
O2 saturation (%)						
a	98.2±0.2	98.3±0.1	98.1±0.1	98.2±0.1	98.1±0.1	98.2±0.1
v	71.8±1.9	80.4±2.1*	91.8±1.7	93.0±1.2	94.9±0.4	94.8±0.5
O2 content (ml l-1)						
a	184±3	184±3	186±2	185±3	186±2	185±3
v	134±5	146±5*	170±5	171±3	176±3	175±3
Leg VO2 (ml min-1)						
	15±2	20±3	22±4	29±8	24±5	29±5
Heart rate (beats min-1)						
	63±3	63±3	69±3	70±2	74±3	72±3
FVP (mmHg)						
	4.3±0.8	2.4±0.5*	4.5±0.8	3.1±0.8*	4.7±0.7	3.5±0.7

a: femoral arterial; v. femoral venous. Significant difference from CON within same condition: *P < 0.05.

Online Table 1B. Blood variables at rest and during ACh infusion

Blood variable	Older					
	Rest		ACh 1		ACh 2	
	CON	SILD	CON	SILD	CON	SILD
PO2 (mmHg)						
a	85±6#	77±5#	82±4#	79±6#	83±4#	79±6#
v	40±2	42±2	55±3#	53±4#	65±4	60±3#
Hemoglobin (g dl-1)						
a	12.9±0.3#	12.3±0.4*#	12.8±0.4#	12.4±0.4*#	12.8±0.4#	12.4±0.4*#
v	12.7±0.3#	12.3±0.4*#	12.8±0.4#	12.2±0.3*#	12.7±0.4#	12.4±0.4#
O2 saturation (%)						
a	95.6±0.7#	94.5±0.8*#	95.8±0.5#	94.3±1.0*#	95.8±0.5#	94.3±1.0*#
v	73.0±2.8	75.4±2.9	85.7±2.3	84.2±3.7#	90.1±0.9#	89.4±1.7#
O2 content (ml l-1)						
a	167±4#	159±5*#	167±5#	159±5*#	167±5#	159±5*#
v	124±4	125±6#	148±4#	139±6#	156±5#	151±4*#
Leg VO2 (ml min-1)						
	13±3	19±5	17±3	16±4	23±4	19±6
Heart rate (beats min-1)						
	62±3	64±5	64±3	66±4	68±3	68±4
FVP (mmHg)						
	3.9±0.6	3.7±0.7	4.5±0.6	4.2±0.7	4.7±0.7	4.4±0.6

a: femoral arterial; v. femoral venous. Significant difference from CON within same condition: *P < 0.05.

significant difference from young within same condition: #P < 0.05.

Online Table 2A. Blood variables at rest and during SNP infusion

Blood variable	Young			
	Rest		SNP	
	CON	SILD	CON	SILD
PO2 (mmHg)				
a	106±3	109±4	108±2	102±2*
v	40±2	43±2	70±6	68±1
Hemoglobin (g dl-1)				
a	13.9±0.3	13.7±0.2	14.1±0.3	13.8±0.2
v	13.5±0.3	13.2±0.2	14.1±0.4	13.4±0.2
O2 saturation (%)				
a	98.2±0.1	98.3±0.2	98.3±0.1	98.1±0.1
v	73.4±1.8	75.4±3.0	92.8±0.7	93.6±0.4
O2 content (ml l-1)				
a	185±4	184±3	188±4	183±3
v	134±5	135±6	178±5	170±3
Leg VO2 (ml min-1)				
	14±2	18±2	13±3	20±3
Heart rate (beats min-1)				
	64±3	60±3	70±3	71±3
FVP (mmHg)				
	3.5±0.7	2.8±0.8*	3.2±0.7	2.7±0.7*

a: femoral arterial; v. femoral venous. Significant difference from CON within same condition: *P < 0.05.

Online Table 2B. Blood variables at rest and during SNP infusion

Blood variable	Older			
	Rest		SNP	
	CON	SILD	CON	SILD
PO2 (mmHg)				
a	85±4	76±5*#	84±4	78±5*
v	38±1	41±2	59±2	57±3
Hemoglobin (g dl-1)				
a	12.8±0.4#	12.3±0.4#	12.7±0.3#	12.2±0.3#
v	12.5±0.3#	12.2±0.3#	12.7±0.4#	12.2±0.3#
O2 saturation (%)				
a	96.1±0.5#	94.4±1.0*#	96.2±0.6#	94.8±0.7*#
v	70.1±1.2#	73.4±3.1#	88.8±1.4#	87.8±2.2#
O2 content (ml l-1)				
a	167±5#	157±4*#	167±4#	157±4*#
v	119±3#	121±6	153±4#	146±4#
Leg VO2 (ml min-1)				
	16±4	16±4	18±4	19±4
Heart rate (beats min-1)				
	61±3	60±4	68±3	69±4
FVP (ml min-1 mmHg-1)				
	4.1±0.5	3.9±0.7*	3.7±0.6	4.3±0.9

a: femoral arterial; v. femoral venous. Significant difference from CON within same condition: *P < 0.05; significant difference from young within same condition: #P < 0.05.
